# Supplementary material for: A diagnostic classifier for gene expression-based identification of early Lyme disease
Source: Commun Med (Lond). 2022 Jul 22;2:92. doi: 10.1038/s43856-022-00127-2 (PMC9306241; doi:10.1038/s43856-022-00127-2)
Supplement: Supplementary file 1 — Supplementary Information [file 43856_2022_127_MOESM1_ESM.pdf]

SUPPLEMENTARY INFORMATION

A Diagnostic Classifier for Gene Expression-Based Identification of Early Lyme Disease

Venice Servellita[1#], Jerome Bouquet[1#], Alison Rebman[2], Ting Yang[2], Erik Samayoa[1], Steve Miller[1], Mars Stone[3], Marion Lanteri[3], Michael Busch[3], Patrick Tang[4], Muhammad Morshed[5], Mark J. Soloski[2], John Aucott[2] and Charles Y Chiu[1,6*]

[1]Department of Laboratory Medicine, University of California, San Francisco, CA, USA

[2]Lyme Disease Research Center, Division of Rheumatology, Department of Medicine, Johns Hopkins School of Medicine, Baltimore, MD, USA

[3]Blood Systems Research Institute, San Francisco, CA, USA

[4]Sidra Medical and Research Center, Doha, Qatar

[5]British Columbia Centre for Disease Control, Vancouver, British Columbia, Canada

[6]Department of Medicine, Division of Infectious Diseases, University of California, San Francisco, CA, USA

[+] E-mail : charles.chiu@ucsf.edu (CYC)

[#] These authors contributed equally

*Correspondence to:
Charles Chiu, MD/PhD
Department of Laboratory Medicine and Medicine, Division of Infectious Diseases
University of California, San Francisco, San Francisco, CA
e-mail: charles.chiu@ucsf.edu
tel: (415) 420-4463

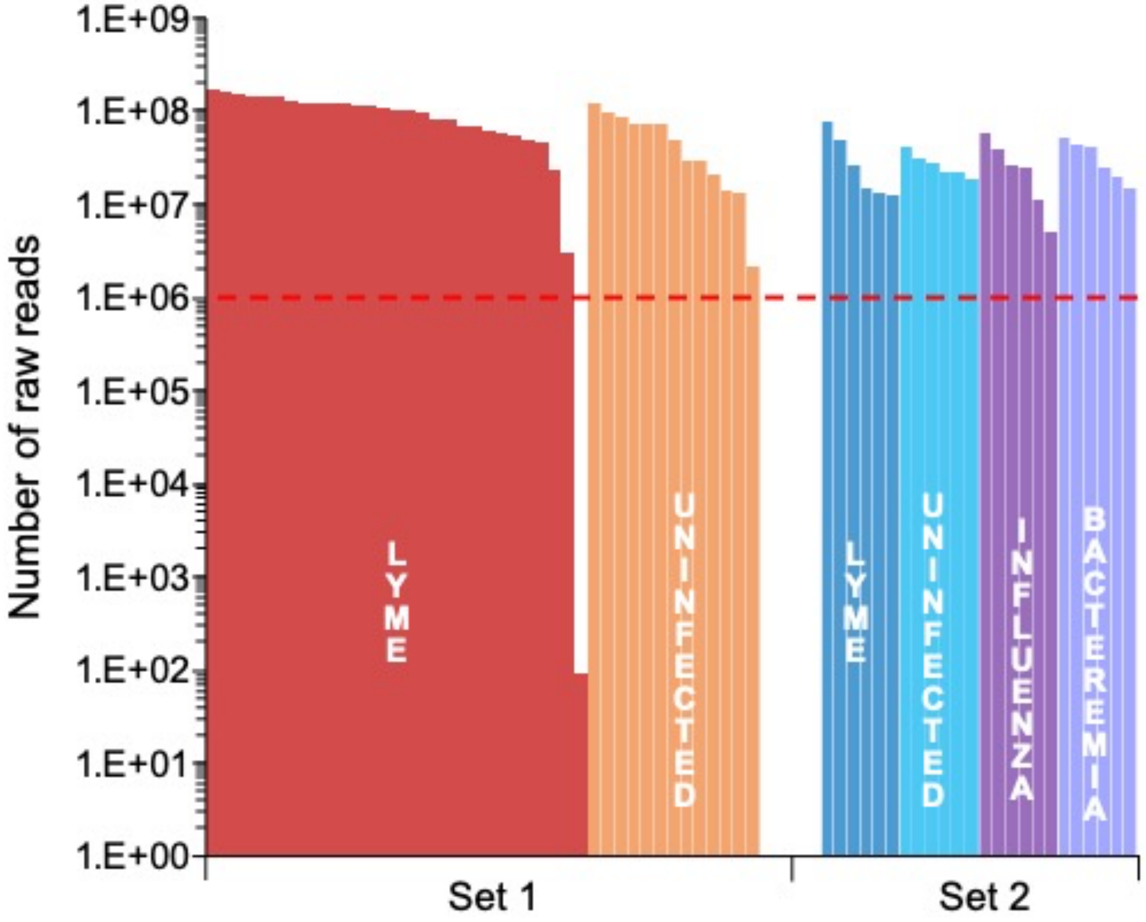

Supplementary Figure 1. Transcriptome RNA-Seq read counts.

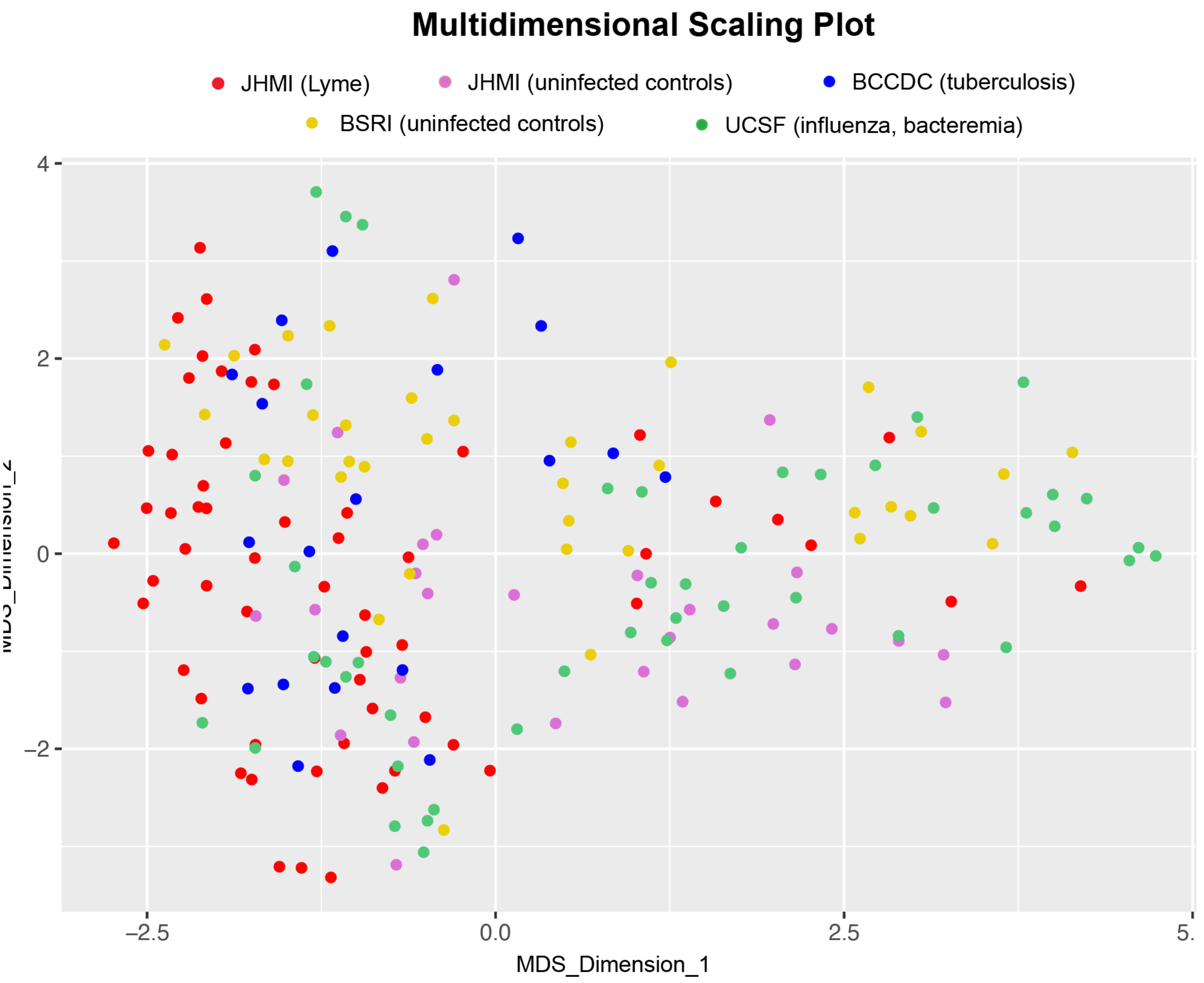

Supplementary Figure 2. Multidimensional scaling (MDS) plot of targeted RNA expression data for the training set samples (n=137) used for machine learning-based analyses. Each point represents a single sample and is color-coded by geographic site of origin. The distance between 2 points reflects the leading log fold-change, or the average of the largest absolute log fold-change between each pair of samples for the genes that best distinguish the pair of samples. No clustering based on geographic site is observed. Abbreviations: JHMI, Johns Hopkins Medical Institute; BCCDC, British Columbia Centers for Disease Control; BSRI, Blood Systems Research Institute; UCSF, University of California, San Francisco.

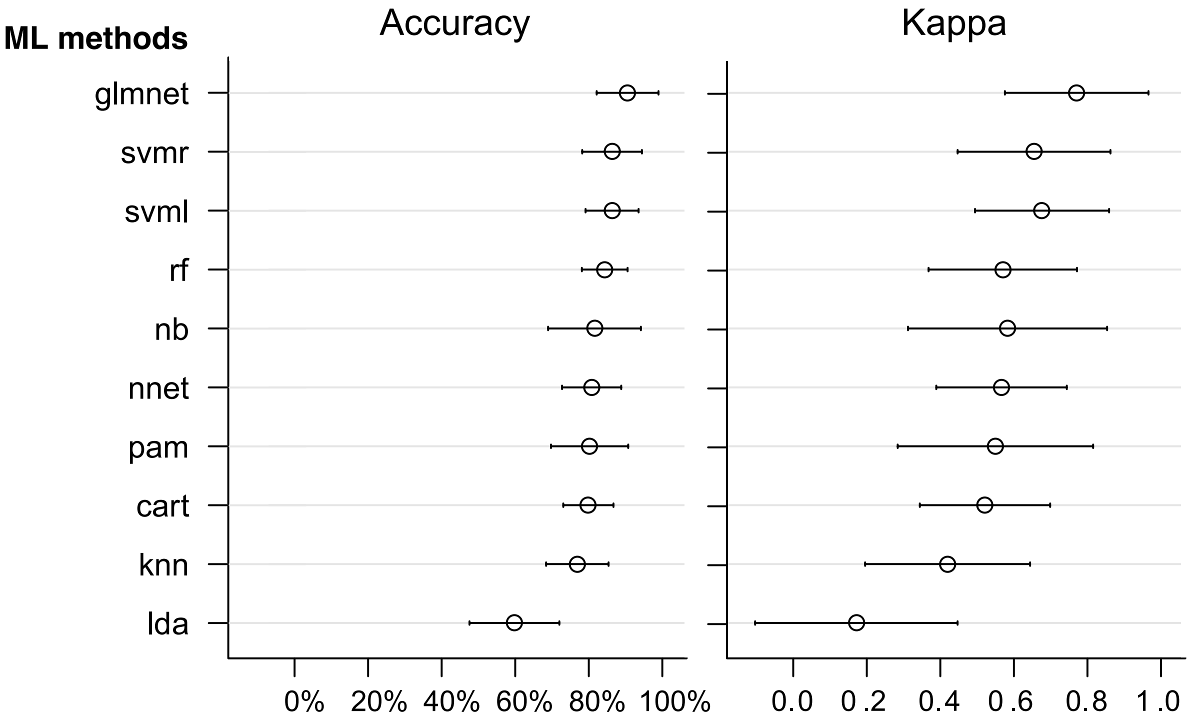

Supplementary Figure 3. Comparison of the performance of different 10 machine learning algorithms for Lyme disease classification based on training set data. The model showing the best performance, or highest AUC-ROC value, uses the "glmnet" algorithm. The error bars represent the standard deviation of the accuracy based on the results of 10-fold cross-validation. Abbreviations: AUC-ROC, area under the curve – receiver operating characteristic; glmnet, lasso and elastic-net regularized generalized linear models; xgbt, eXtreme Gradient Boosting; rf, random forest; svm, support vector machine; nnet, neural network; pam, partitioning around medoids; knn, k-nearest neighbors; lda, linear discriminant analysis; rpart, recursive partitioning and regression trees.
