# Supplementary material for: A diagnostic classifier for gene expression-based identification of early Lyme disease
Source: Commun Med (Lond). 2022 Jul 22;2:92. doi: 10.1038/s43856-022-00127-2 (PMC9306241; doi:10.1038/s43856-022-00127-2)
Supplement: Supplementary file 6 — Description of Additional Supplementary Files [file 43856_2022_127_MOESM6_ESM.pdf]

Description of Additional Supplementary Files

File Name: Supplementary Data 1

Description: Metadata for the 263 clinical samples included in this study.

File Name: Supplementary Data 2

Description: Gene panels used for targeted RNA expression sequencing (TREx) runs.

File Name: Supplementary Data 3

Description: Composition of the 31-gene Lyme disease classifier panel.

File Name: Supplementary Data 4

Description: Source data for this study.
