# Supplementary material for: A diagnostic classifier for gene expression-based identification of early Lyme disease
Source: Commun Med (Lond). 2022 Jul 22;2:92. doi: 10.1038/s43856-022-00127-2 (PMC9306241; doi:10.1038/s43856-022-00127-2)
Supplement: Supplementary file 8 — Reporting Summary [file 43856_2022_127_MOESM8_ESM.pdf]

nature portfolio

Corresponding author(s): Charles Chiu

Last updated by author(s): Mar 24, 2022

Reporting Summary

Nature Portfolio wishes to improve the reproducibility of the work that we publish. This form provides structure for consistency and transparency in reporting. For further information on Nature Portfolio policies, see our Editorial Policies and the Editorial Policy Checklist.

Statistics

For all statistical analyses, confirm that the following items are present in the figure legend, table legend, main text, or Methods section.

n/a	Confirmed	
☐	☒	The exact sample size (*n*) for each experimental group/condition, given as a discrete number and unit of measurement
☐	☒	A statement on whether measurements were taken from distinct samples or whether the same sample was measured repeatedly
☐	☒	The statistical test(s) used AND whether they are one- or two-sided<br>*Only common tests should be described solely by name; describe more complex techniques in the Methods section.*
☒	☐	A description of all covariates tested
☒	☐	A description of any assumptions or corrections, such as tests of normality and adjustment for multiple comparisons
☐	☒	A full description of the statistical parameters including central tendency (e.g. means) or other basic estimates (e.g. regression coefficient) AND variation (e.g. standard deviation) or associated estimates of uncertainty (e.g. confidence intervals)
☐	☒	For null hypothesis testing, the test statistic (e.g. *F*, *t*, *r*) with confidence intervals, effect sizes, degrees of freedom and *P* value noted<br>*Give P values as exact values whenever suitable.*
☒	☐	For Bayesian analysis, information on the choice of priors and Markov chain Monte Carlo settings
☒	☐	For hierarchical and complex designs, identification of the appropriate level for tests and full reporting of outcomes
☒	☐	Estimates of effect sizes (e.g. Cohen's *d*, Pearson's *r*), indicating how they were calculated

Our web collection on statistics for biologists contains articles on many of the points above.

Software and code

Policy information about availability of computer code

Data collection	Peripheral blood mononuclear cells (PBMCs) were isolated from fresh collected whole blood in EDTA tubes using Ficoll (Ficoll-Paque Plus, GE Healthcare) and total RNA was extracted from 107 PBMCs using TRIzol reagent (Life Technologies). Messenger RNA was isolated with the Oligotex mRNA mini kit (Qiagen). The Scriptseq RNA-Seq library preparation kit (Epicentre) was used to generate the RNA-Seq libraries according to the manufacturer's protocol. Libraries were sequenced as 100 bp paired-end reads on a HiSeq 2000 instrument (Illumina).<br><br>Targeted RNA enrichment sequencing was done by incorporating an anchored multiplex PCR technique. PBMC samples (~1 million cells) were extracted using using Zymo DirectZol RNA Miniprep Kit with on-column DNase following the manufacturer's instructions. Reverse transcription was performed using the Illumina TruSeq Targeted RNA Expression Kit on 50 ng of RNA according to the manufacturer's instructions. A custom panel of oligoucleotides representing the genes of interest was designed and ordered using the Illumina DesignStudio platform. This pool of oligonucleotides, each attached to a small RNA sequencing primer (smRNA) binding site, was used to hybridize, extend and ligate the second strand of cDNA from targeted genes of interest. A total of 35 cycles of amplification were then performed using primers with a complementary smRNA sequence. The resulting libraries were sequenced on an Illumina MiSeq to a depth of ~2,500 reads per sample per gene.
Data analysis	Paired-end reads were mapped to the human genome (hg19), followed by annotation of exons and calculation of FPKM (fragments per kilobase of exon per million fragments mapped) values for all 25,278 expressed genes with version 2 of the TopHat/Cufflinks pipeline. Differential expression of genes was calculated using the variance modeling at the observational level transformation, which applies precision weights to the matrix count, followed by linear modeling with the Limma package. Genes were considered to be differentially expressed when the change was greater than 1.5-fold, the p-value was 0.05, and the adjusted p-value (or false discovery rate, FDR) was 0.1%.<br><br>Expression counts per sample per gene was calculated on the instrument using MiSeq reporter targeted RNA workflow software (revision C). Briefly, following demultiplexing and FASTQ file generation, reads from each sample were normalized in R and then aligned locally against references corresponding to targeted regions of interest using a banded Smith-Waterman algorithm .

The k-nearest neighbor classification with leave-one-out cross validation algorithm (KNNXV), as implemented on Genepattern, was used on the set of DEGs identified by RNA-Seq-based transcriptome profiling, using a k of 3, signal-to-noise ratio feature selection, Euclidean distance, and by iteratively decreasing the number of features until reaching maximum accuracy. Class prediction performance using ROC metric on targeted RNA sequencing read count results was tested using the glmnet and caret packages in R for 10 different machine learning methods at default parameters.

For manuscripts utilizing custom algorithms or software that are central to the research but not yet described in published literature, software must be made available to editors and reviewers. We strongly encourage code deposition in a community repository (e.g. GitHub). See the Nature Portfolio guidelines for submitting code & software for further information.

Data

Policy information about availability of data

All manuscripts must include a data availability statement. This statement should provide the following information, where applicable:

- Accession codes, unique identifiers, or web links for publicly available datasets
- A description of any restrictions on data availability
- For clinical datasets or third party data, please ensure that the statement adheres to our policy

All sequencing data in this study were submitted to the database of Genotypes and Phenotypes (dbGaP) (read count tables, raw FASTQ files for transcriptome sets 1 and 2 accession number phs002794.v1.p1). Metadata for the 263 clinical samples included in this study are provided in Supplementary Data 1. Source data used to generate the main figures are provided in Supplementary Data 4. Code used to reproduce the ML analysis for LDC model prediction and feature selection has been deposited in a Zenodo repository (doi: 10.5281/zenodo.5987532).

Field-specific reporting

Please select the one below that is the best fit for your research. If you are not sure, read the appropriate sections before making your selection.

☒ Life sciences ☐ Behavioural & social sciences ☐ Ecological, evolutionary & environmental sciences

For a reference copy of the document with all sections, see nature.com/documents/nr-reporting-summary-flat.pdf

Life sciences study design

All studies must disclose on these points even when the disclosure is negative.

Sample size	The study comprised a total of 263 samples from 218 subjects (Table 1 and Supplementary Table 1). The 218 subjects included 94 Lyme disease patients, 66 infected "non-Lyme" controls with influenza (n=36), tuberculosis (n=9), and other bacteremia (n=21), and 58 uninfected asymptomatic controls.
Data exclusions	No experimental data were excluded. There were Inclusion and exclusion data for samples as described in the manuscript.
Replication	A subset of samples used in transcriptome profiling were also used for targeted RNA-sequencing for gene pruning and as part of the training set. All samples in the test set were sequenced and analyzed only a single time.
Randomization	Training and test sets were randomly selected. After ensuring that the training set consisted entirely of samples from laboratory-confirmed ("Lyme seropositive") Lyme disease patients and that no prior analyses had been performed on the independent test set, 137 and 63 samples were assigned to the training and test sets, respectively, at an approximately 2:1 (68.5%:31.5%) ratio.
Blinding	Researchers were blinded to disease status during sample processing and analysis of the transcriptome data.

Reporting for specific materials, systems and methods

We require information from authors about some types of materials, experimental systems and methods used in many studies. Here, indicate whether each material, system or method listed is relevant to your study. If you are not sure if a list item applies to your research, read the appropriate section before selecting a response.

Materials & experimental systems

n/a	Involved in the study	
☒	☐	Antibodies
☒	☐	Eukaryotic cell lines
☒	☐	Palaeontology and archaeology
☒	☐	Animals and other organisms
☐	☒	Human research participants
☒	☐	Clinical data
☒	☐	Dual use research of concern

Methods

n/a	Involved in the study	
☒	☐	ChIP-seq
☒	☐	Flow cytometry
☒	☐	MRI-based neuroimaging

Human research participants

Policy information about studies involving human research participants

Population characteristics	See Supplemental Table 1 for a summary of metadata associated with the 263 samples included in this study.
Recruitment	All 94 Lyme disease subjects included in this study presented with a physician documented EM of ≥ 5cm and either concurrent flu-like symptoms that included at least one of the following: fever, chills, fatigue, headache and/or new muscle or joint pains or dissemination of the EM rash to multiple skin locations. Controls (n=28) were enrolled from the same physician practice as cases. PBMC samples from 57 patients diagnosed with other infections and 22 controls (asymptomatic blood donors), were collected in San Francisco, California. PBMC samples from 19 adults, 9 patients diagnosed with tuberculosis using an interferon-gamma release assay (Oxford Immunotec T-SPOT.TB) and 10 uninfected controls, were collected in Vancouver, Canada.
Ethics oversight	Patient enrollment, chart review, collection of clinical samples, and analysis of clinical samples by transcriptomic profiling or targeted RNA sequencing were done under protocols approved by the Institutional Review Boards of Johns Hopkins University and the University of California, San Francisco.

Note that full information on the approval of the study protocol must also be provided in the manuscript.
